# Co-Administration of Melatonin Effectively Enhances the Therapeutic Effects of Pioglitazone on Mesenchymal Stem Cells Undergoing Indoxyl Sulfate-Induced Senescence through Modulation of Cellular Prion Protein Expression

**DOI:** 10.3390/ijms19051367

**Published:** 2018-05-04

**Authors:** Yong Seok Han, Sang Min Kim, Jun Hee Lee, Sang Hun Lee

**Affiliations:** 1 Soonchunhyang Medical Science Research Institute, Soonchunhyang University, Soonchunhyang University Seoul Hospital, Seoul 04401, Korea; format7000@naver.com (Y.S.H.); sangno1120@gmail.com (S.M.K.); 2 Neuroregeneration and Stem Cell Programs, Institute for Cell Engineering, Department of Neurology, The Johns Hopkins University School of Medicine, Baltimore, MD 21287, USA; 3 Department of Pharmacology and Toxicology, University of Alabama at Birmingham School of Medicine, Birmingham, AL 35294, USA; j-school@hanmail.net

**Keywords:** indoxyl sulfate, mesenchymal stem cells, cell senescence, pioglitazone, melatonin

## Abstract

Background: Mesenchymal stem cells (MSCs) are a promising source for regenerative medicine. However, their therapeutic potential in patients with chronic kidney disease (CKD) is restricted by the presence of uremic toxins. To address this limitation, we explored the protective effect of melatonin and pioglitazone on MSCs undergoing senescence induced by the uremic toxin, indoxyl sulfate (IS). Methods: MSC senescence was induced by IS, and the therapeutic effects of melatonin and pioglitazone were identified. The expression of cellular prion protein (PrP^C^) was suppressed by transfection of MSCs with prion protein gene (*PRNP*) siRNA. Subsequently, these cells were used to study the protective effects of melatonin and pioglitazone against IS-induced senescence; Results: The IS-induced senescence of MSCs was significantly reduced by co-treatment with melatonin and pioglitazone compared to treatment with melatonin or pioglitazone alone. In the presence of IS, the reduced MSC proliferation was rescued by co-treatment with melatonin and pioglitazone. Melatonin and pioglitazone enhanced the expression of peroxisome proliferator-activated receptor-γ (PPAR-γ) in MSCs, which resulted in the augmentation of PrP^C^ level. The inhibitory effect of the co-treatment with melatonin and pioglitazone on IS-induced senescence in MSCs was blocked by the knockdown of *PRNP*. In addition, the restorative effect of the co-treatment on the reduced MSC proliferation induced by IS was also blocked by the knockdown of *PRNP*. These findings indicate that co-treatment with melatonin and pioglitazone protected MSCs from uremic toxin-induced senescence through the regulation of the PPAR-γ-PrP^C^ axis. Conclusions: Our study suggests that co-treatment of MSCs with melatonin and pioglitazone may represent a novel strategy for the development of MSC-based therapies for patients with CKD.

## 1. Introduction

Chronic kidney disease (CKD) is a growing public health issue that affects between 8% and 16% of the global population [1,2] and accounts for more than $80 billion of the US Medicare budget [3,4]. CKD results from the progressive loss of kidney function and the distortion of homeostasis in the body, leading to the improper clearance of toxic metabolites and organic waste solutes. Such protein-bound uremic toxins cause numerous complications in the body, including cardiovascular disease, weakened bones, damage to the central nervous system, persistent systemic inflammation, and irreversible damage to the kidney that requires dialysis or kidney transplantation [5,6,7,8]. Among the different types of uremic toxins, indoxyl sulfate (IS) and p-cresol sulfate have been extensively reported to cause adverse outcomes in patients with CKD [9]. IS is a toxic protein metabolized in the liver by using tryptophan-derived indole produced by tryptophanase present in intestinal bacteria such as *Escherichia coli* [10]. Although IS is normally cleared via renal proximal tubular secretion by healthy kidneys, it is not effectively removed in patients with CKD, even with the currently available dialysis technologies [11].

A growing body of evidence suggests that stem cell regenerative therapy provides an attractive option for the treatment of CKD [12,13]. Mesenchymal stem cells (MSCs) have emerged as a promising source for regenerative medicine for many reasons: MSCs exist in adult tissues from various sources, are able to self-renew and differentiate into several types of specialized cells, such as osteoblasts, chondrocytes, adipocytes, and tenocytes, and are expandable in vitro, maintaining a stable genome [14]. MSC therapy, if properly applied, contributes to cellular repair and the amelioration of renal injury in patients with CKD [15,16]. However, as damaged tissues lead to pathophysiological conditions, such as low nutrients, limited oxygen, and inflammation, the survival of transplanted MSCs into the targeted tissues is drastically decreased. Consequently, the main limiting factor of MSC therapy is that, as MSCs age, they undergo only a limited number of divisions before ceasing to proliferate [17]. Moreover, MSC senescence has been associated with diminished differentiation potential, which reduces the intended therapeutic applicability [18]. IS, a ubiquitous uremic toxin circulating in patients with CKD, is known to cause senescence and, subsequently, apoptosis of cells in the body via reactive oxygen species (ROS) generation in endothelial cells [17,19,20]. Such effects result in a reduction of cellular proliferation and wound repair abilities, which complicates the recuperation and recovery of patients with CKD [21]. In addition, IS promotes renal fibrosis by inducing the expression and phosphorylation of p53 via ROS production [22]. In patients with CKD, uremic toxins, including IS, significantly limit the therapeutic efficacy of MSCs through the induction of MSC senescence, which prevents the proliferation and maintenance of MSC viability when injected into the patient. Accumulating evidence has suggested that MSC senescence is likely to be pivotal for the clinical application of stem cell therapy in patients with CKD, and therapeutic approaches to MSC senescence have not been fully defined.

Deleterious adverse complications related to CKD and the limited feasibility of currently known therapies serve as a motivation to seek a novel, more effective therapeutic strategy for the prevention or delay of renal injury and of the progression to CKD [23,24,25]. Previous studies have suggested that patients with CKD experience sleep impairments, such as obstructive sleep apnea and restless leg syndrome, and that progression to CKD or end-stage renal disease (ESRD) may be correlated with the development of various sleep disorders [26,27]. Similarly, our recent study showed that melatonin (*N*-acetyl-5-methoxy tryptamine) secreted by the pineal gland reduces MSCs senescence induced by uremic toxins commonly accumulated in patients with CKD, which suggested that it may offer a potential intervention to enhance MSC-derived therapy for the prevention of CKD progression [26,28]. Just as melatonin is thought to possess a wide variety of biological activities, such as anti-apoptotic, anti-inflammatory, autophagy-modulating, and antitumor effects [29,30,31,32], pioglitazone, a peroxisome proliferator-activated receptor-γ (PPARγ) agonist, is known to prevent apoptosis, improve cell differentiation capacity, and promote cell proliferation [33,34]. In addition, pioglitazone is thought to increase stem cell viability in patients with various diseases [35,36] and is known to produce potent therapeutic effects, via the upregulation of growth factors and the reduction of anti-apoptotic effects, and improved differentiation effects [36,37]. Insights into MSC cell viability after a combination treatment of melatonin, an over-the-counter drug widely used clinically as a sleeping aid, and pioglitazone, a drug with proven potency in MSC viability, may provide us with better options to improve the functionality and viability of MSCs and enable the practical use of these drugs in patients with CKD. Therefore, to explore whether co-treatment with melatonin and pioglitazone prevented uremic toxin-induced senescence in MSCs, we investigated the protective effect of melatonin and pioglitazone against IS-induced senescence in MSCs.

## 2. Results

### 2.1. Pioglitazone and Melatonin Protected from Uremic Toxin-Induced Senescence

**Figure 1:**
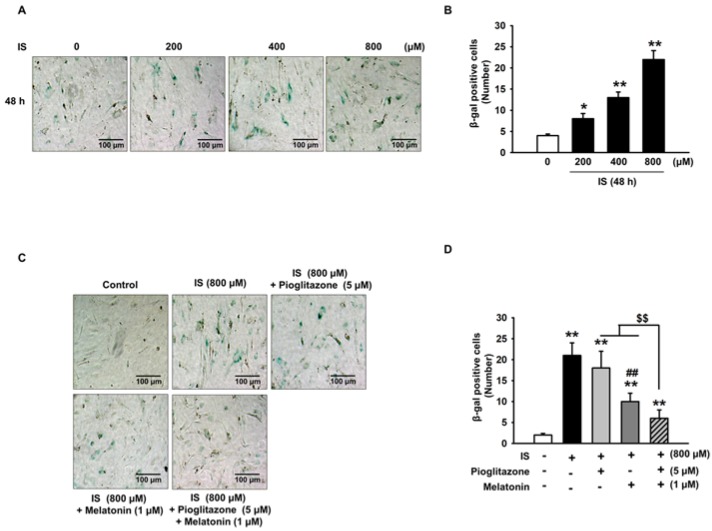
Inhibition of indoxyl sulfate-induced cell senescence by melatonin and pioglitazone. (**A**) Mesenchymal stem cells (MSCs) were exposed to indoxyl sulfate (IS), and representative images of senescence-associated beta-galactosidase (SA-β-gal) activity stain are shown. Scale bar = 100 μm; (**B**) Number of SA-β-gal positive cells (*n* = 3). The values represent the mean ± standard error of the mean (SEM). * *p* < 0.05 and ** *p* < 0.01 vs. control (Analysis of variance (ANOVA), using Dunnett’s post-hoc test); (**C**) SA-β-gal activity stain assay representative image of IS-exposed MSCs treated with pioglitazone and melatonin. Scale bar = 100 μm; (**D**) number of SA-β-gal positive cells (*n* = 3). The values represent the mean ± SEM. ** *p* < 0.01 vs. control, ## *p* < 0.01 vs. IS only, $$ *p* < 0.01 vs. pretreatment with pioglitazone or melatonin and IS (ANOVA, using Tukey’s post-hoc test).

**Figure 2:**
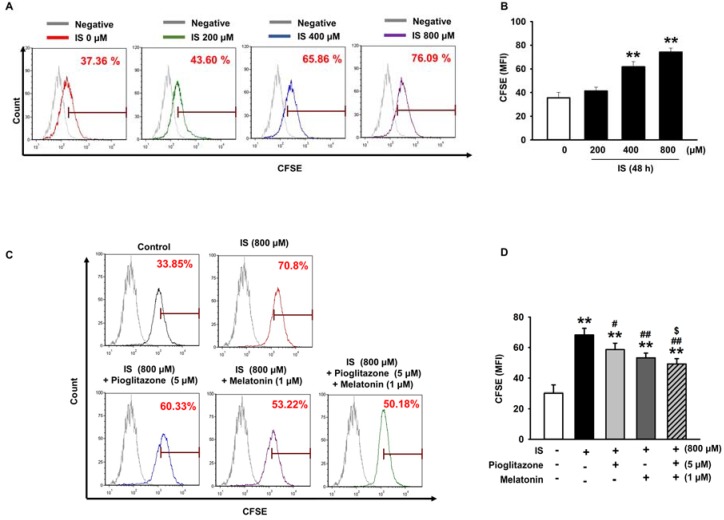
Pioglitazone and melatonin restore cell proliferation reduced by IS. (**A**,**B**) The images show carboxyfluorescein succinimidyl ester (CFSE)-labeled MSCs exposed to IS (0–800 µM) for 48 h; cell proliferation was assessed by fluorescence-activated cell sorting (FACS) analysis of the dilution of CFSE in the same number of viable cells (*n* = 3). The values represent the mean ± SEM; ** *p* < 0.01 vs. control (ANOVA, using Dunnett’s post-hoc test); (**C**) the graphs show CFSE-labeled IS-exposed MSCs pretreated with pioglitazone and melatonin; (**D**) FACS analysis of the dilution of CFSE in the same number of viable cells (*n* = 3). The values represent the mean ± SEM; ** *p* < 0.01 vs. control, # *p* < 0.05 and ## *p* < 0.01 vs. IS only, $ *p* < 0.05 vs. pretreatment with pioglitazone or melatonin and IS (ANOVA, using Tukey’s post-hoc test).

The MSCs were exposed to various concentrations of IS for 48 h. To investigate the effect on MSCs senescence after IS treatment, we performed staining for senescence-associated beta-galactosidase (SA-β-gal) activity in MSCs. The results showed that IS treatment increased the number of MSCs that stained positive for SA-β-gal activity and that IS increased senescence of MSCs in a concentration-dependent manner (Figure 1A,B). We tested various concentrations and showed that 5 µM pioglitazone and 1 µM melatonin promoted the highest growth rates of MSCs (Figure S1). Consequently, we investigated the inhibitory effects of pioglitazone and melatonin on MSC senescence induced by IS. The combination therapy of pioglitazone and melatonin significantly reduced IS-induced increase of SA-β-gal activity (Figure 1C,D). Moreover, co-treatment with pioglitazone and melatonin also protected MSCs from senescence induced by serum isolated from a murine model of CKD (Figure S2). In addition, IS was verified, by using a Carboxyfluorescein succinimidyl ester (CFSE) assay, to decrease the proliferative capacity of MSCs in a concentration-dependent manner at 48 h (Figure 2A,B). However, the combination therapy with pioglitazone and melatonin significantly increased the proliferative capacity of MSCs. These results indicate that pioglitazone and melatonin counteracted the reduction of MSC proliferation and the induction of cell senescence caused by IS.

### 2.2. Pioglitazone and Melatonin Regulate Cell Senescence-Related Protein Expression

**Figure 3:**
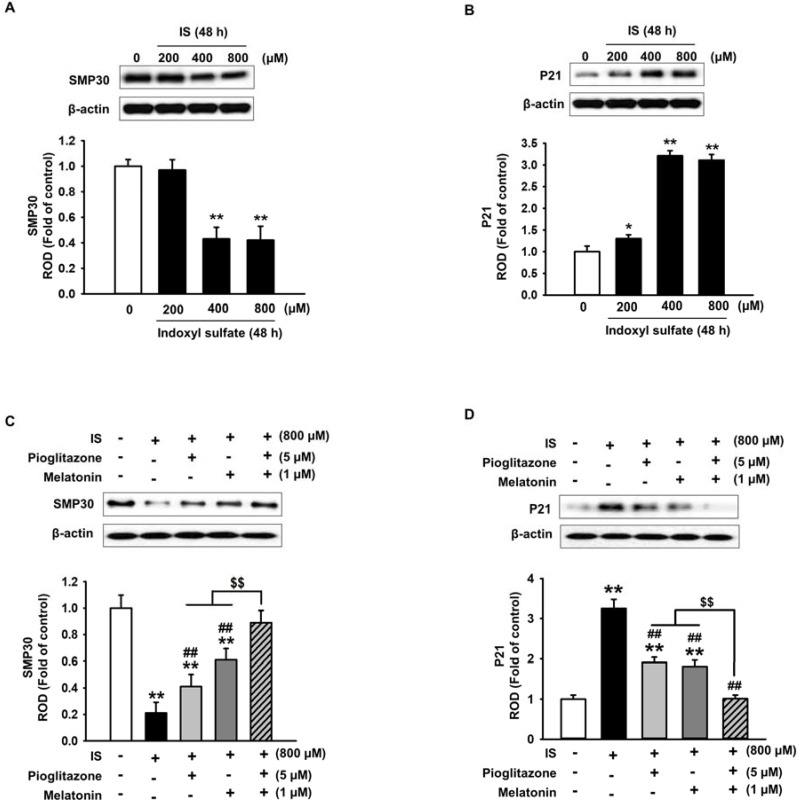
Pioglitazone and melatonin regulate SMP30 and P21 expression. (**A**,**B**) Western blotting of SMP30 and p21 in MSCs treated with various concentrations of IS (0–800 µM) (*n* = 3). The values represent the mean ± SEM; * *p* < 0.05 and ** *p* < 0.01 vs. control (ANOVA, using Dunnett’s post-hoc test); (**C**,**D**) western blotting of SMP30 and p21 in IS-exposed MSCs pretreated with pioglitazone and melatonin (*n* = 3). The values represent the mean ± SEM; ** *p* < 0.01 vs. control, ## *p* < 0.01 vs. IS only, $$ *p* < 0.01 vs. pretreatment with pioglitazone or melatonin and IS (ANOVA, using Tukey’s post-hoc test).

As shown in Figure 2, the combination treatment of pioglitazone and melatonin ameliorated the IS-induced reduction in MSC proliferation. Therefore, we investigated the changes in the expression of cell senescence marker proteins after IS-induced aging of MSCs. The expression of aging-related protein senescence marker protein 30 (SMP30) decreases with aging and the expression of p21 increases with aging [38,39]. In our experiments, IS induced a decrease in SMP30 expression and an increase in p21 expression in a concentration-dependent manner (Figure 3A,B), which confirmed previous results that IS induces cell senescence. However, pioglitazone and melatonin ameliorated the IS-induced reduction in SMP30 expression (Figure 3C) and inhibited the increase in p21 (Figure 3D). These results, in addition to those shown in Figure 1, indicate that a combination treatment of pioglitazone and melatonin inhibited IS-induced senescence of MSCs to a greater extent than the individual treatments.

### 2.3. Pioglitazone and Melatonin Increase Cellular Prion Protein Expression

**Figure 4:**
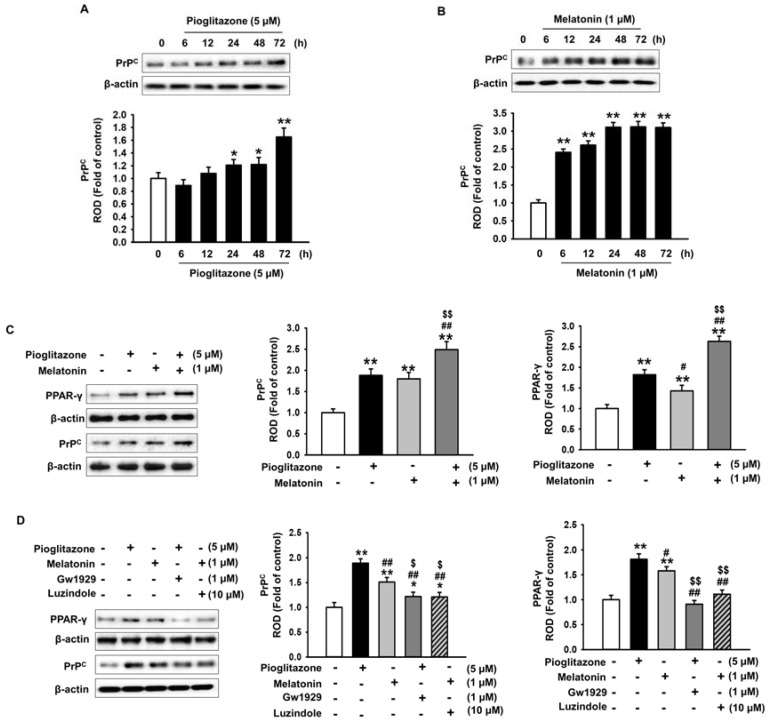
Pioglitazone and melatonin regulate cellular prion protein expression. (**A**) Western blotting of cellular prion protein (PrP^C^) in MSCs pretreated with pioglitazone (5 µM) for 0, 6, 12, 24, 48, and 72 h (*n* = 3). The values represent the mean ± SEM; * *p* < 0.05 and ** *p* < 0.01 vs. control (ANOVA, using Dunnett’s post-hoc test); (**B**) western blotting analysis of PrP^C^ in MSCs pretreated with melatonin (1 µM) for 0, 6, 12, 24, 48, and 72 h (*n* = 3). The values represent the mean ± SEM; ** *p* < 0.01 vs. control (ANOVA, using Dunnett’s post-hoc test); (**C**) western blotting of PPAR-γ and PrP^C^ in MSCs pretreated with pioglitazone (5 µM) and melatonin (1 µM) for 72 h (*n* = 3). The values represent the mean ± SEM; ** *p* < 0.01 vs. control, # *p* < 0.05 and ## *p* < 0.01 vs. pioglitazone only, $$ *p* < 0.01 vs. melatonin only (ANOVA, using Tukey’s post-hoc test); (**D**) western blotting of PPAR-γ and PrP^C^ in PPAR-γ antagonist (GW1929)- or melatonin receptor inhibitor (luzindole)-pre-treated MSCs treated with pioglitazone and melatonin (*n* = 3). The values represent the mean ± SEM; * *p* < 0.05 and ** *p* < 0.01 vs. control, # *p* < 0.05 and ## *p* < 0.01 vs. IS only, $ *p* < 0.05 and $$ *p* < 0.01vs. pretreatment with pioglitazone or melatonin and treatment with IS (ANOVA, using Tukey’s post-hoc test).

We performed an experiment to determine how pioglitazone and melatonin inhibited cell senescence induced by urotoxicity. Treatment with pioglitazone and melatonin individually increased PPAR-γ and cellular prion protein (PrP^C^) expression in a time-dependent manner (Figure 4A,B). In addition, the combination of pioglitazone and melatonin increased PrP^C^ expression (Figure 4C). Furthermore, pioglitazone was verified, through the use of a PPAR-γ antagonist, to regulate PrP^C^ expression through PPAR-γ; similarly, melatonin was verified, by using a melatonin receptor inhibitor, to regulate PrP^C^ expression through melatonin receptors (Figure 4D). These results confirmed that PPAR-γ was involved in the expression of PrP^C^.

### 2.4. Inhibition of Senescence through the Expression of Cellular Prion Protein

**Figure 5:**
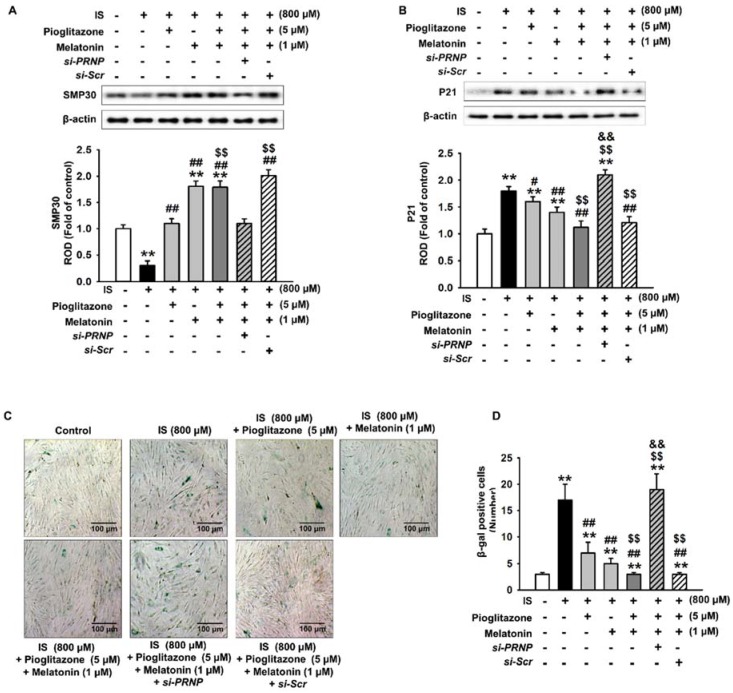
Pioglitazone and melatonin protect MSCs from urotoxin-induced cell senescence through PrP^C^. (**A**,**B**) Western blotting of SMP30 and p21 in MSCs transfected with *PRNP* siRNA, treated with pioglitazone and melatonin (*n* = 3). The values represent the mean ± SEM; ** *p* < 0.01 vs. control, # *p* < 0.05 and ## *p* < 0.01 vs. IS only, $$ *p* < 0.01 vs. pretreatment with pioglitazone or melatonin and IS (ANOVA, using Tukey’s post-hoc test); (**C**) representative images of the SA-β-gal activity stain assay. Scale bar = 100 μm; (**D**) number of SA-β-gal positive cells (*n* = 3). The values represent the mean ± SEM; ** *p* < 0.01 vs. control, ## *p* < 0.01 vs. IS only, $$ *p* < 0.01 vs. pretreatment with pioglitazone or melatonin and IS, && *p* < 0.01 vs. pretreatment with pioglitazone and melatonin and treatment with IS (ANOVA, using Tukey’s post-hoc test).

**Figure 6:**
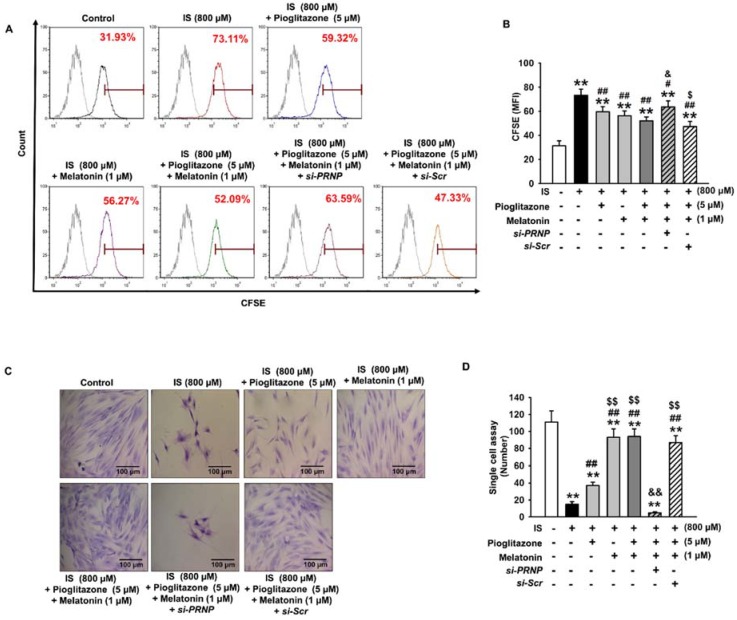
Pioglitazone and melatonin restore cell proliferation via PrP^C^. (**A**) Images of CFSE-labeled MSCs and IS-exposed MSCs treated with pioglitazone and melatonin or *PRNP* siRNA-transfected MSCs; (**B**) FACS analysis of samples containing the dilution of CFSE in the same number of viable cells (*n* = 3). The values represent the mean ± SEM; ** *p* < 0.01 vs. control, # *p* < 0.05 and ## *p* < 0.01 vs. IS only, $ *p* < 0.05 vs. pretreatment with pioglitazone or melatonin and IS, & *p* < 0.05 vs. pretreatment with pioglitazone and melatonin and IS (ANOVA, using Tukey’s post-hoc test); (**C**) single-cell cultures of IS-exposed MSCs treated with pioglitazone and melatonin or *PRNP* siRNA-transfected MSCs were stained with Giemsa after 10 days of growth. Scale bar = 100 μm; (**D**) images of the number of cells per well in 96-well plates (*n* = 3). The values represent the mean ± SEM; ** *p* < 0.01 vs. control, ## *p* < 0.01 vs. IS only, $$ *p* < 0.01 vs. pretreatment with pioglitazone or melatonin and IS, && *p* < 0.01 vs. pretreatment with pioglitazone and melatonin and treatment with IS (ANOVA, using Tukey’s post-hoc test).

We also examined the role of PrP^C^ expression in the inhibitory effects of the combination treatment on cell senescence. In bone marrow-derived MSCs isolated from normal and CKD mice, cellular senescence was significantly higher in CKD MSCs than in MSCs. The expression of PrPC was also significantly lower in CKD MSCs than in normal MSCs (Figure S3). In human adipose tissue-derived MSCs, our results showed that pioglitazone and melatonin did not inhibit cell senescence induced by IS when the expression of PrP^C^ was inhibited, as shown by the reversed expression of SMP30 and P21 (Figure 5A,B). In addition, when the expression of PrP^C^ was inhibited, pioglitazone and melatonin did not protect against the decrease in cell senescence induced by the urotoxin (Figure 5C,D). Furthermore, when PrP^C^ expression was suppressed, pioglitazone and melatonin did not protect against the reduction of cell proliferation induced by the urotoxin (Figure 6). These results indicate that pioglitazone and melatonin protected MSCs from senescence induced by urotoxicity through the expression of PrP^C^.

## 3. Discussion

The main objective of our study was to test whether a combination treatment with two existing drugs, namely, melatonin and pioglitazone, resulted in an enhanced outcome compared with treatments with either drug alone. In our study, we confirmed that the uremic toxin IS caused MSC senescence and repressed cellular proliferation. Moreover, we confirmed that the combination treatment of pioglitazone and melatonin more effectively prevented IS-induced MSC senescence and death via PrP^C^ upregulation compared with either melatonin or pioglitazone alone.

The use of MSCs for cell-based therapies is a potentially viable clinical intervention for patients with CKD for whom no treatments are available to prevent the progression of their disease to end-stage kidney failure [40,41]. Advances in the field of regenerative medicine over the past decade have allowed the development of stem cell therapies suitable for kidney repair. MSCs, known for their autocrine and paracrine anti-inflammatory, cell proliferative, and anti-senescence effects, are shown not only to improve kidney’s endogenous reparative capacity, but also to enhance kidney transplantation efficacy via the regulation of various cytokines responsible for immunomodulation [42]. Such desirable characteristics present a promising solution targeted to patients with CKD. However, the clinical applicability of MSCs is significantly inhibited by the high concentration of uremic toxins present in the blood of the patients, as these toxins induce senescence and apoptosis of MSCs [17]. More specifically, IS is one of the most ubiquitous protein-bound uremic toxins in patients with CKD that may inhibit the effective application of MSCs. Studies have shown that IS significantly inhibited cell proliferation, altered procalcific phenotypes, and caused senescence of MSCs, thus reducing their potential viability [43,44]. To confirm our assumption that IS induces MSC senescence, we treated MSCs with various concentrations of IS. First, we evaluated the number of β-gal positive cells in response to increased IS concentration. Our results suggest that the number of β-gal positive cells significantly increased in a concentration-dependent manner after IS treatment, which indicated an increase in MSC senescence in response to IS. Previous studies have utilized senescence marker protein 30 (SMP 30) and apoptotic mediator protein p21 as important protein markers for cell senescence [45,46]. SMP30 is known to play an essential role in intracellular Ca^2+^ homeostasis, ascorbic acid biosynthesis, oxidative stress, and detoxification, and a decrease in this protein has been correlated with cell aging. The protein p21 is a potent cyclin-dependent kinase inhibitor (CKI) known to promote cell cycle arrest in response to many stimuli. In our study, we used western blotting to determine SMP30 and p21 levels and found that SMP30 expression decreased and p21 expression increased in IS-treated MSCs. In addition, our CFSE staining results supported this, showing that IS treatment resulted in a high fluorescent intensity in MSCs, denoting a reduced capacity for cell proliferation. These results suggest that IS induced MSC senescence and decreased MSC proliferation; thus, interventions are necessary to counteract the accumulated IS in patients with CKD and achieve the successful use of MSCs.

Accumulated evidence has suggested that pioglitazone is a highly desirable therapeutic to increase the effectiveness of MSC therapy in patients with CKD. For example, Shinmura et al. reported that the transplantation of pioglitazone-pretreated bone-marrow MSCs improved the efficiency of MSC cardiomyogenic transdifferentiation and ultimately repaired heart tissues in a model of myocardial infarction [35]. In addition, the pretreatment of MSCs with pioglitazone was shown to improve the feasibility of MSC transplantation in heart disease, with minimal side effects, enhancing the transmission of MSCs to the core of injured tissue and their differentiation to novel cells of the tissue [35,47]. In addition to pioglitazone, our previous research proved that melatonin rescued MSCs from p-cresol-induced senescence via increased cell proliferation and catalase activity, ultimately preventing cell autophagy [28]. Given the high applicability of the two drugs in the clinical setting, we sought to determine the feasibility of a combination treatment of MSCs and to examine if it was more effective than the treatments with either drug alone. Our results suggest that both pioglitazone and melatonin, when incubated individually with MSCs, inhibited cellular senescence and subsequently increased cellular proliferation. When incubated with MSCs pretreated with IS, both melatonin and pioglitazone rescued MSCs from the senescence effects of IS, as shown via β-gal analysis; however, the best therapeutic results were obtained with a concurrent treatment with pioglitazone and melatonin. Similarly, the concurrent treatment of melatonin and pioglitazone rescued the IS-induced decrease in SMP30 senescence protein marker and reduced the increase of p21, as shown in our western blot analysis. The results of CSFE staining showed that pioglitazone and melatonin rescued the cell proliferation capacity suppressed by IS. The cellular prion protein PrP^C^ is a ubiquitous glycoprotein anchored to the outer leaflet of the plasma membrane in the raft domain through a glycosylphosphatidylinositol (GPI) moiety. Although abnormalities in prion proteins are commonly known to induce neurodegenerative conditions such as transmissible spongiform encephalopathies, several studies have identified crucial roles of normal PrP^C^ in cell cycle and proliferation [48,49,50,51]. Consequently, researchers discovered that PrP^C^ was ubiquitously expressed throughout the body and had a critical role in cell proliferation, cell cycle progression, and cell protection. Previously, our team reported evidence that melatonin regulated MSCs via the upregulation of PrP^C^ [52]. Specifically, melatonin-mediated overexpression of PrP^C^ decreased apoptosis of MSCs under oxidative stress conditions through the regulation of the apoptosis-associated proteins BCL-2, BAX, PARP-1, and caspase-3, and the immunomodulatory PrP^C^ axis. This suggested that PrP^C^ in melatonin-treated MSCs could provide a therapeutic strategy for vessel regeneration. In search of such evidence, we investigated whether our pioglitazone and melatonin combination treatment exerted similar protective and proliferative effects via PPAR-γ and PrP^C^ expression. The positive effects of melatonin and pioglitazone treatment demonstrated by our results were proven to be regulated via a PrP^C^-dependent pathway. Western blotting analysis indicated that pioglitazone, as well as melatonin, increased the expression of PPAR-γ and PrP^C^. In addition, the simultaneous treatment with inhibitors of both drugs nullified the increased level of PrP^C^ induced by each drug, indicating that both drugs were directly related to and responsible for the modulation of the PPAR-γ-PrP^C^ axis. Then, we studied whether the inhibition PrP^C^ in MSCs, through gene silencing, nullified the reduced senescence and cell proliferation effects observed via western blot analysis of SMP 30 and p21 expression [45,46], SA-β-gal staining, and the single cell proliferation assay.

In the current study, we examined whether the combination treatment of melatonin and pioglitazone was effective in protecting the MSCs from cell senescence induced by the uremic toxin IS. More specifically, we were able to confirm that these therapeutic effects were driven by the PrP^C^ pathway: in fact the combination treatment exerted anti-aging effects and increased the proliferation of MSCs through the upregulation of PrP^C^ expression. In a previous study, it was reported that in patients with stage 4–5 CKD, the serum concentration of IS was significantly higher than normal [53]. Under uremic toxin-induced pathophysiological conditions, the transplantation efficacy of MSC significantly decreased [54]. MSCs isolated from an animal model of CKD have various dysfunctions, such as reduced proliferation and suppressed cytokine secretion [55]. Our findings indicate that the combination treatment with melatonin and pioglitazone protected against uremic toxin-induced cellular senescence and rescued MSC proliferation through the regulation of the PPAR-γ-PrP^C^ axis. Although the effect of the combination treatment with melatonin and pioglitazone in MSC-based therapies requires further investigation in in vivo models of CKD, this study suggests that the combination treatment might offer a novel strategy for the development of MSC-based therapeutics. Furthermore, targeting of PPAR-γ and PrP^C^ may assist the development of uremic toxin-induced senescence-resistant MSCs for MSC applications in patients with CKD.

In summary, we provide in vitro evidence that the concurrent treatment of pioglitazone and melatonin could help prevent MSC senescence induced by the accumulation of IS in patients with CKD and may be a novel combination treatment to improve the stem cell-based therapy of CKD.

## 4. Materials and Methods

### 4.1. Mesenchymal Stem Cell Culture

The MSCs used in the experiments were derived from human adipose tissue and purchased from the American Type Culture Collection (ATCC; Manassas, VA, USA). The MSCs used were confirmed to be free of pathogens and mycoplasmas. The expression of MSCs surface markers CD73 and CD105 was also confirmed, and their adipogenic and osteogenic differentiation potentials were demonstrated. MSCs were cultured in Minimum Essential Medium Eagle (MEM) α essential medium (Gibco BRL, Gaithersburg, MD, USA) supplemented with 10% fetal bovine serum (FBS), 100 U/mL of penicillin, and 100 µg/mL of streptomycin, and grown in an incubator in 5% CO_2_ atmosphere at 37 °C.

### 4.2. BrdU Incorporation Assay

The cell proliferation parameters were assessed using a cell proliferation 5-bromo-2′-deoxyuridine (BrdU) ELISA colorimetric kit (Roche, Mannheim, Germany). To perform ELISA, 100 μg/mL BrdU was added to cultured MSCs and incubated at 37 °C for 6 h. Subsequently, 1 M H_2_SO_4_ was added to stop the reaction. The light absorbance at 450 nm of the samples was measured by using an ELISA reader (BMG labtech, Ortenberg, Germany). The experiments were performed in triplicate.

### 4.3. Beta-Galactosidase Staining Assay

Senescence was assessed through the measurement of the percentage of cultured cells that were positively stained for senescence-associated beta-galactosidase (SA-β-gal) activity, as described previously. The cells were washed twice with phosphate-buffered saline (PBS) and fixed with 2% formaldehyde/0.2% glutaraldehyde (Sigma-Aldrich, St. Louis, MO, USA). The fixed cells were incubated at 37 °C in a CO_2_-free environment for 12 h with a β-gal staining solution (1 mg/mL X-Gal, 40 mM citric acid-sodium phosphate buffer, 150 mM NaCl, 2 mM MgCl_2_, 5 mM potassium ferrocyanide, and 5 mM potassium ferricyanide; pH 6.0; Sigma-Aldrich). The stained (blue, positive) and unstained (negative) cells were counted by using phase contrast microscopy (Nikon, Tokyo, Japan) in five independent cultures. The experiments were performed in triplicate.

### 4.4. Western Blot Analysis

Total cellular proteins (20 μg protein) were separated by using 10% sodium dodecyl sulfate-polyacrylamide gel electrophoresis. The separated proteins were transferred to a nitrocellulose membrane. The membranes were washed with TBST (10 mM Tris-HCl (pH 7.6), 150 mM NaCl, and 0.05% Tween-20), blocked with 5% skim milk for 2 h, and then incubated with primary antibodies specific to SMP30, P21, PrP^C^, and β-actin (Santa Cruz Biotechnology, Dallas, TX, USA). After incubation of the membranes with the primary antibodies, peroxidase-conjugated secondary antibodies (Santa Cruz Biotechnology) were added. The protein bands were detected by using enhanced chemiluminescence (ECL) reagents (Amersham Biosciences, Little Chalfont, UK), in a dark room. The experiments were performed in triplicate.

### 4.5. CFSE Staining Assay

The MSCs were washed once in PBS, diluted in 1 mL of MEM α medium supplemented with 5% FBS, and stained with 10 μM CFSE (Molecular Probes, Eugene, OR, USA) for 10 min at room temperature. The cells were protected from light during staining. After staining, the cells were washed three times with PBS, and sthe tained MSCs were cultured in an incubator with 5% CO_2_ at 37 °C. After treatment with IS, pioglitazone, and melatonin, the samples were analyzed using the green (FL1) channel of the flow cytometer. The analytical measurements showed that CFSE-positive staining decreased when cell proliferation was enhanced, and increased when cell proliferation was suppressed. The experiments were performed in triplicate.

### 4.6. siRNA Transfection

MSCs were cultured to 70% confluence in culture plates. The cells were transfected for 48 h with Lipofectamine 2000 reagent (Thermo Fisher Scientific, Rockford, IL, USA) with mixed *PRNP*-specific SMART pool siRNAs (100 nM/L). The transfected MSCs were used in experiments after replacement with siRNA-free growth media.

### 4.7. Single-Cell Cultivation Assay

MSCs in culture were resuspended as single cells by using trypsin. Using a limited-dilution assay, MSCs were adjusted to 1 cell per 100 μL. The suspended MSCs were seeded in 96-well plates at 100 μL per well, and one cell per well was cultured. After 12 h, the MSCs attached to the plate were treated with IS, pioglitazone, and melatonin. The MSCs were grown in an incubator with 5% CO_2_ at 37 °C for 10 days. The experiments were performed in triplicate.

### 4.8. Statistical Analyses

All data are presented as the mean ± standard error of the mean (SEM). All experiments were analyzed by one-way analysis of variance (ANOVA). Comparisons of three or more groups were made by using Dunnett’s or Tukey’s post-hoc test. A *p* value < 0.05 was considered statistically significant.

## Supplementary Materials

Supplementary materials can be found at http://www.mdpi.com/1422-0067/19/5/1367/s1.
