# Supplementary material for: Co-Administration of Melatonin Effectively Enhances the Therapeutic Effects of Pioglitazone on Mesenchymal Stem Cells Undergoing Indoxyl Sulfate-Induced Senescence through Modulation of Cellular Prion Protein Expression
Source: Int J Mol Sci. 2018 May 4;19(5):1367. doi: 10.3390/ijms19051367 (PMC5983612; doi:10.3390/ijms19051367)
Supplement: Supplementary file 1 [file ijms-19-01367-s001.pdf]

Supplementary Materials

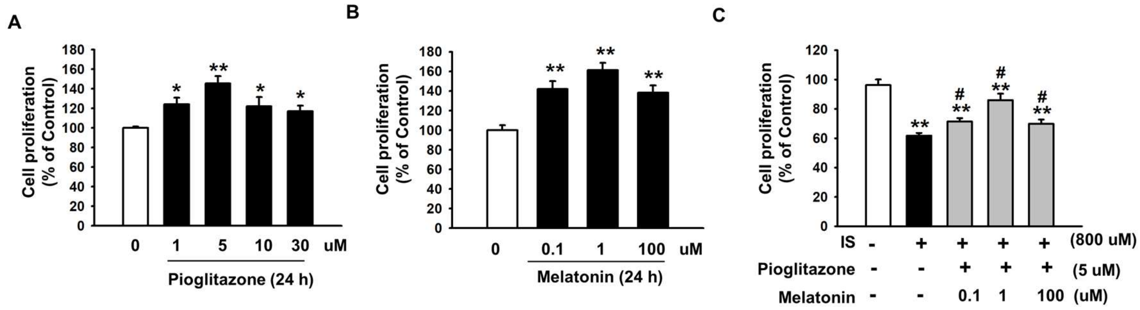

Figure S1. The effect of pioglitazone and melatonin on MSC proliferation. (**A**) After treatment of MSCs with pioglitazone (0, 1, 5, 10, and 30 μM) for 24 h, cell proliferation was assessed. The values represent the mean ± SEM. * $p < 0.05$ and ** $p < 0.01$ vs. non-treatment; (**B**) After treatment of MSCs with melatonin (0, 0.1, 1, 100 μM) for 24 h, cell proliferation was assessed. The values represent the mean ± SEM. ** $p < 0.01$ vs. non-treatment; (**C**) After exposure to indoxyl sulfate (IS; 800 μM), cell proliferation was assessed after pretreatment with pioglitazone (5 μM) and melatonin (0, 0.1, 1, 100 μM) for 24 h. The values represent the mean ± SEM. ** $p < 0.01$ vs. non-treatment, # $p < 0.05$ vs. MSCs treated IS.

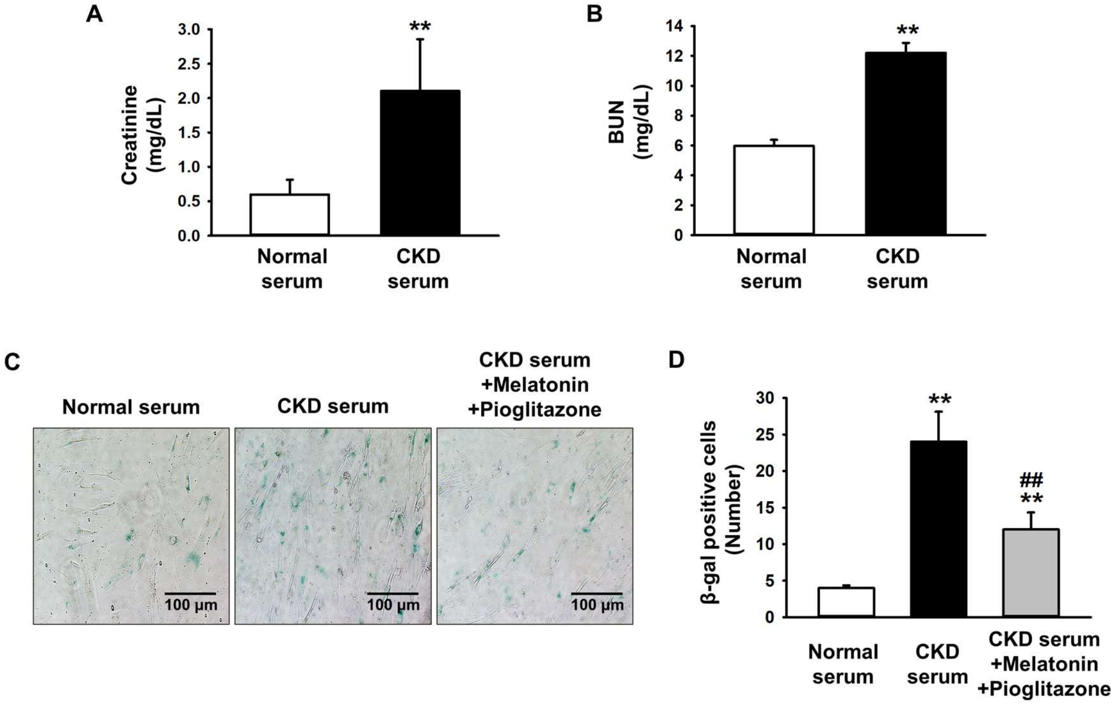

Figure S2. Effect of serum isolated from chronic kidney disease (CKD) mouse model on MSC senescence. (**A**) The concentration of creatinine in serum isolated from normal (normal serum) and CDK (CKD serum) mice. The values represent the mean ± SEM. ** $p < 0.01$ vs. normal serum; (**B**) The concentration of blood urea nitrogen (BUN) in normal and CDK serum. The values represent the mean ± SEM. ** $p < 0.01$ vs. normal serum; (**C**) Images of senescence-associated beta-galactosidase (SA-β-gal) staining in MSCs pretreated with melatonin and pioglitazone after exposure to CKD serum; (**D**) The number of SA-β-gal positive cells. The values represent the mean ± SEM. ** $p < 0.01$ vs. normal serum, ## $p < 0.01$ vs. CKD serum.

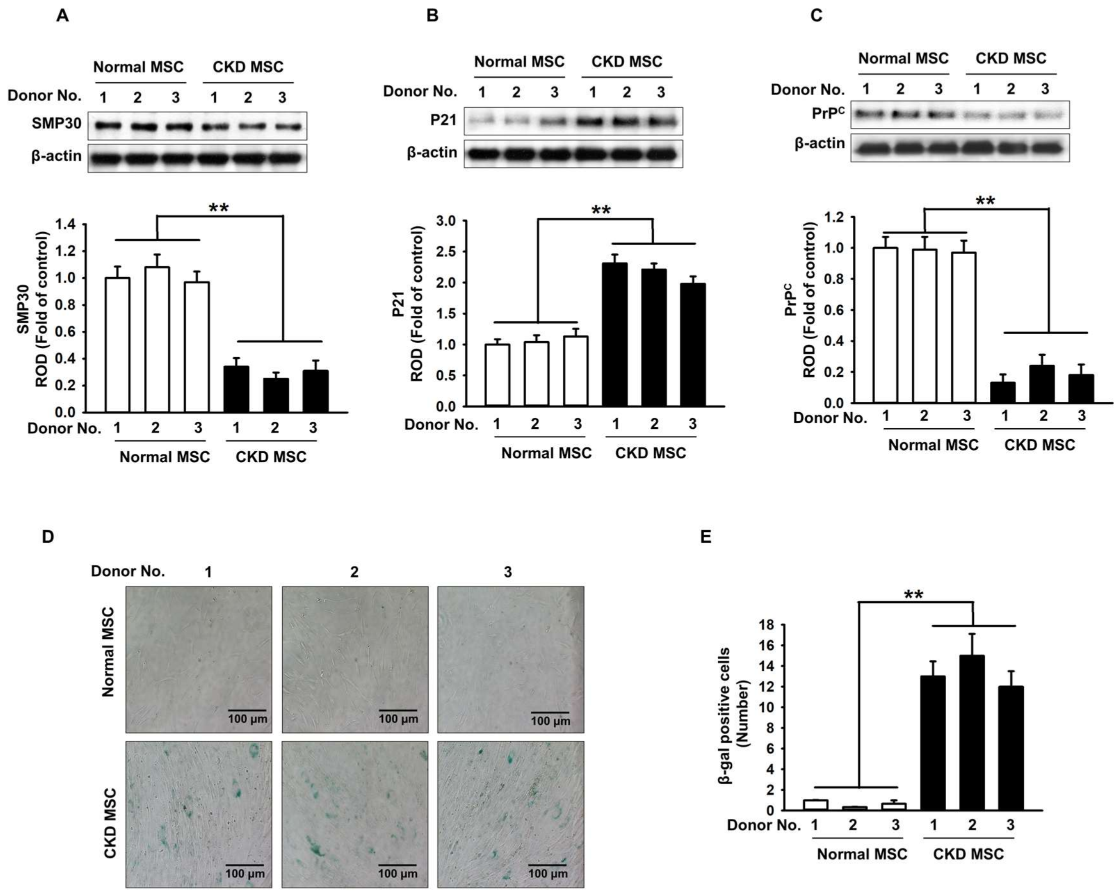

Figure S3. Assessment of senescence in bone marrow-derived MSCs isolated from CKD mice. (**A**–**C**) The expressions of SMP30 (**A**), P21 (**B**), and PrP^{C} (**C**) in bone marrow-derived MSCs isolated from normal (normal MSC; $n = 3$) and CKD (CKD MSC; $n = 3$) mice. The values represent the mean ± SEM. ** $p < 0.01$ vs. Normal MSC; (**D**) Images of senescence-associated beta-galactosidase (SA-β-gal) staining in normal ($n = 3$) and CKD ($n = 3$) MSCs; (**E**) The number of SA-β-gal positive cells. The values represent the mean ± SEM. ** $p < 0.01$ vs. normal MSCs.
